# Viability and thresholds for treatment of extremely preterm infants: survey of UK neonatal professionals

**DOI:** 10.1136/archdischild-2020-321273

**Published:** 2021-04-29

**Authors:** Lydia Mietta Di Stefano, Katherine Wood, Helen Mactier, Sarah Elizabeth Bates, Dominic Wilkinson

**Affiliations:** 1 Faculty of Medicine, Nursing and Health Sciences, Monash University, Clayton, Victoria, Australia; 2 Newborn Care, John Radcliffe Hospital, Oxford, UK; 3 Neonatology, Princess Royal Maternity Hospital, Glasgow, UK; 4 School of Medicine, Dentistry & Nursing, University of Glasgow, Glasgow, UK; 5 Department of Paediatrics and Neonatology, Great Western Hospitals NHS Foundation Trust, Swindon, UK; 6 Oxford Uehiro Centre for Practical Ethics, University of Oxford, Oxford, UK

**Keywords:** ethics, neonatology, resuscitation, palliative care

## Abstract

**Background:**

Decisions about treatments for extremely preterm infants (EPIs) born in the ‘grey zone’ of viability can be ethically complex. This 2020 survey aimed to determine views of UK neonatal staff about thresholds for treatment of EPIs given a recently revised national Framework for Practice from the British Association of Perinatal Medicine.

**Methods:**

The online survey requested participants indicate the lowest gestation at which they would be willing to offer active treatment and the highest gestation at which they would withhold active treatment of an EPI at parental request (their lower and upper thresholds). Relative risks were used to compare respondents’ views based on profession and neonatal unit designation. Further questions explored respondents’ conceptual understanding of viability.

**Results:**

336 respondents included 167 consultants, 127 registrars/fellows and 42 advanced neonatal nurse practitioners (ANNPs). Respondents reported a median grey zone for neonatal resuscitation between 22^+1^ and 24^+0^ weeks’ gestation. Registrars/fellows were more likely to select a lower threshold at 22^+0^ weeks compared with consultants (Relative Risk (RR)=1.37 (95% CI 1.07 to 1.74)) and ANNPs (RR=2.68 (95% CI 1.42 to 5.06)). Those working in neonatal intensive care units compared with other units were also more likely to offer active treatment at 22^+0^ weeks (RR=1.86 (95% CI 1.18 to 2.94)). Most participants understood a fetus/newborn to be ‘viable’ if it was possible to survive, regardless of disability, with medical interventions accessible to the treating team.

**Conclusion:**

Compared with previous studies, we found a shift in the reported lower threshold for resuscitation in the UK, with greater acceptance of active treatment for infants <23 weeks’ gestation.

**What is already known on this topic?**

- Decision-making for extremely preterm infants is medically complex and ethically controversial.
- There have been improvements in outcomes for the most immature infants over time.
- A 2019 UK Framework for Practice indicates that stabilisation may be considered from 22^+0^ weeks’ gestation following risk assessment and discussion with parents.

**What this study adds?**

- The majority of UK neonatal clinicians would be prepared to offer active treatment for extremely premature infants at 22^+1^ weeks’ gestation.
- The lower threshold, but not the upper threshold of the grey zone has shifted in the UK.
- There are some differences in views between professions and those who work in neonatal intensive care compared with other units.

## Introduction

Preterm birth contributes to preventable deaths and health consequences in survivors, including learning, motor, visual and hearing disabilities.^1 2^ The greatest burden is in extremely preterm infants (EPIs) born before 28 weeks of pregnancy.^3^ Earlier gestations are associated with worse outcomes, however other factors such as fetal growth, sex and ethnicity as well as multiple pregnancy, maternal demographics and medical interventions all impact on prognosis.^4^

Advances in perinatal care have improved EPI outcomes over time,^2 5^ but such resources are not always available. Outcomes vary both between and within countries, with specialist perinatal centre births associated with improved outcomes <27 weeks’ gestation.^5–8^

Care of women, infants and families around the time of an extremely preterm birth is challenging. Currently, some EPIs survive from 22 weeks’ gestation^9^ however, the risk of longer term neuro-disability is significant. For some infants at extremely high risk of death or severe disability, attempting active stabilisation and survival-focused care (Active Treatment) may not be appropriate and it would be better to offer palliative/comfort-focused care (Palliative Care). For other EPIs, the outcomes are sufficiently good that Active Treatment is potentially in the infant’s best interests. Two thresholds have been described: the ‘lower threshold’, after which Active Treatment is considered ethically optional, and the ‘upper threshold’, after which Active Treatment is considered ethically mandatory. The range of cases between these thresholds is sometimes referred to as the ‘grey zone’.^10–12^

The grey zone boundaries have changed over time. In the 1960s, infants born <28 weeks’ gestation were regarded as ‘previable’.^13^ By the 1990s, approximately 40% of infants born at 24 weeks and receiving active care survived.^9^ UK surveys from 2008 and 2016 found the lower threshold for Active Treatment to be 23 weeks’ gestation.^14 15^

Guidelines have also changed to reflect improvements in EPI outcomes. In 2009, a national framework was published by the British Association of Perinatal Medicine (BAPM) to support extremely preterm birth perinatal care decision-making. The framework indicated Active Treatment should not normally be attempted <23^+0^ weeks and should be attempted >24^+0^ weeks unless severe infant compromise was anticipated.^16^ A revised 2019 BAPM Framework for Practice recommends that Active Treatment from 22^+0^ weeks may be appropriate after risk assessment and consideration of parental views. It emphasises that decision-making must be led by senior obstetric and neonatal staff and in full consultation with parents.^17^

The impact of the 2019 framework on neonatal care in the UK and whether there are differences in its acceptance or uptake are unknown. This study aimed to evaluate views of UK neonatal staff, including consultants, registrars/fellows and ANNPs on Active Treatment for EPIs, enabling assessment of changes over time and comparison with recent international studies.

## Methods

### Participants and procedure

An anonymous online survey was developed to capture views of UK-based neonatal clinicians on decision-making around Active Treatment/Palliative Care for EPIs. Questions on viability and thresholds for Active Treatment were adapted from previous surveys.^15 18^ The survey was piloted and feedback incorporated into the final version (online supplemental file 1).

10.1136/archdischild-2020-321273.supp1 Supplementary data

Participants included consultants, neonatal registrars or fellows and ANNPs who could be involved in decision-making around Active Treatment for EPIs. Recruitment occurred during June–August 2020 via three overlapping sources: the BAPM members’ mailing list (n=1137), the neonatal grid (subspecialist) trainee mailing list (n=152) and an email to ‘REaSoN’ neonatal conference attendees (June 2020) (n=565). Completion was voluntary; all participants indicated informed consent and were asked to complete the survey only once. Two reminder emails were sent to neonatal specialist grid trainees, while BAPM members received a single reminder.

### Design

The survey was developed using Qualtrics.^19^ A scenario of an infant born in fair condition with expected gestational age risk factors was outlined. Participants selected the lowest gestation from a dropdown list (21^+0^–25^+6^ weeks) at which they would offer Active Treatment (ie, resuscitation/stabilisation including intermittent positive pressure ventilation and intubation) at parental request. Participants could indicate that they would always provide Active Treatment for a liveborn infant or provide a free text response. Subsequently, they considered the upper gestational limit at which they would be prepared to provide Palliative Care at parental request.

Further questions explored respondents’ conceptual understanding of the term ‘viability’, including aspects for defining whether a fetus is viable at a particular gestation: the proportion of surviving infants, dependence on technological support and the presence/absence of disability accompanying survival. Agreement with statements about neonatal care developments, their impact on viability and the provision of medical interventions was assessed using Likert scales (online supplemental figure 1).

Optional demographic items captured participants’ age bracket, gender, professional experience and designation of neonatal unit in which they worked: level 1 special care baby unit, level 2 local neonatal unit or level 3 neonatal intensive care unit (NICU).

### Analysis

Consenting respondents who answered ≥1 question were included. Statistical analysis used Microsoft Excel 365 ProPlus and RStudio V.1.1.456. For analysis, respondents selecting ‘other’ for their profession (ie, not consultant, registrar or ANNP) were combined with registrars to form a ‘registrars/fellows’ category. Findings were descriptively presented as frequency (% of respondents for each question) and summarised in tables/graphs. Numerical data from which the graphs are based are presented in the online supplemental tables

Risk ratios were used to examine associations between professional backgrounds (consultants vs registrars/fellows vs ANNPs) and designation of unit worked in (NICU vs other). Standard errors were produced from a binomial generalised linear model with a logarithmic link and fit in R.

## Results

### Sample

A total of 336 eligible responses were received (109 registrars, 167 consultants, 42 ANNPs and 18 other): 61% completed every mandatory question. Two-thirds of participants had >8 years’ experience working with EPIs, and 78% worked in a NICU (table 1).

**Table 1:** Demographic characteristics of respondents

Characteristic	Response	Result, n (%)
Professional level	Consultant	167 (49.7)
	Registrar	109 (32.4)
	ANNP	42 (12.5)
	Other	18 (5.4)
	Total	336

		Consultants	Registrars/ fellows	ANNPs	Total
Years’ experience working with extremely premature infants	0–3	1 (1.0)	10 (14.5)	1 (4.5)	12 (6.0)
	4–7	10 (9.8)	46 (60.5)	1 (4.5)	57 (28.5)
	8–15	30 (29.4)	14 (18.4)	6 (27.3)	50 (25.0)
	16+	61 (59.8)	6 (7.9)	14 (63.6)	81 (40.5)
	Total	102	76	22	200
Neonatal centre usually worked in	NICU	73 (70.2)	70 (90.1)	15 (68.2)	158 (77.8)
	LNU	27 (26.0)	6 (7.8)	7 (31.8)	40 (19.7)
	SCU	4 (3.8)	0 (0.0)	0 (0.0)	4 (2.0)
	Other	0 (0.0)	1 (1.3)	0 (0.0)	1 (0.5)
	Total	104	77	22	203
Gender (n=198)	Female	53 (53.0)	62 (81.6)	21 (95.5)	136 (68.7)
	Male	46 (46.0)	14 (18.4)	1 (4.5)	61 (30.8)
	Other	1 (1.0)	0 (0.0)	0 (0.0)	1 (0.5)
	Total	100	76	22	198
Age (years)	18–30	0 (0.0)	3 (4.0)	0 (0.0)	3 (1.6)
	31–40	20 (20.2)	61 (81.3)	8 (42.1)	89 (46.1)
	41–50	32 (32.3)	5 (6.7)	4 (21.1)	41 (21.2)
	51–60	39 (39.4)	5 (6.7)	7 (36.8)	51 (26.4)
	61–70	7 (7.1)	0 (0.0)	0 (0.0)	7 (3.6)
	71+	1 (1.0)	1 (1.0)	0 (0.0)	2 (1.0)
	Total	99	75	19	193

Note that aside from professional specialty, answering demographic questions was optional.

ANNP, advanced neonatal nurse practitioner; LNU, local neonatal unit; NICU, neonatal intensive care unit; Registars/fellows, registrars and other; SCU, special care unit.

Most respondents were women (69%) and 46% were aged between 31 and 40 years.

### Gestational limits

#### The lower threshold

**Figure 1:**
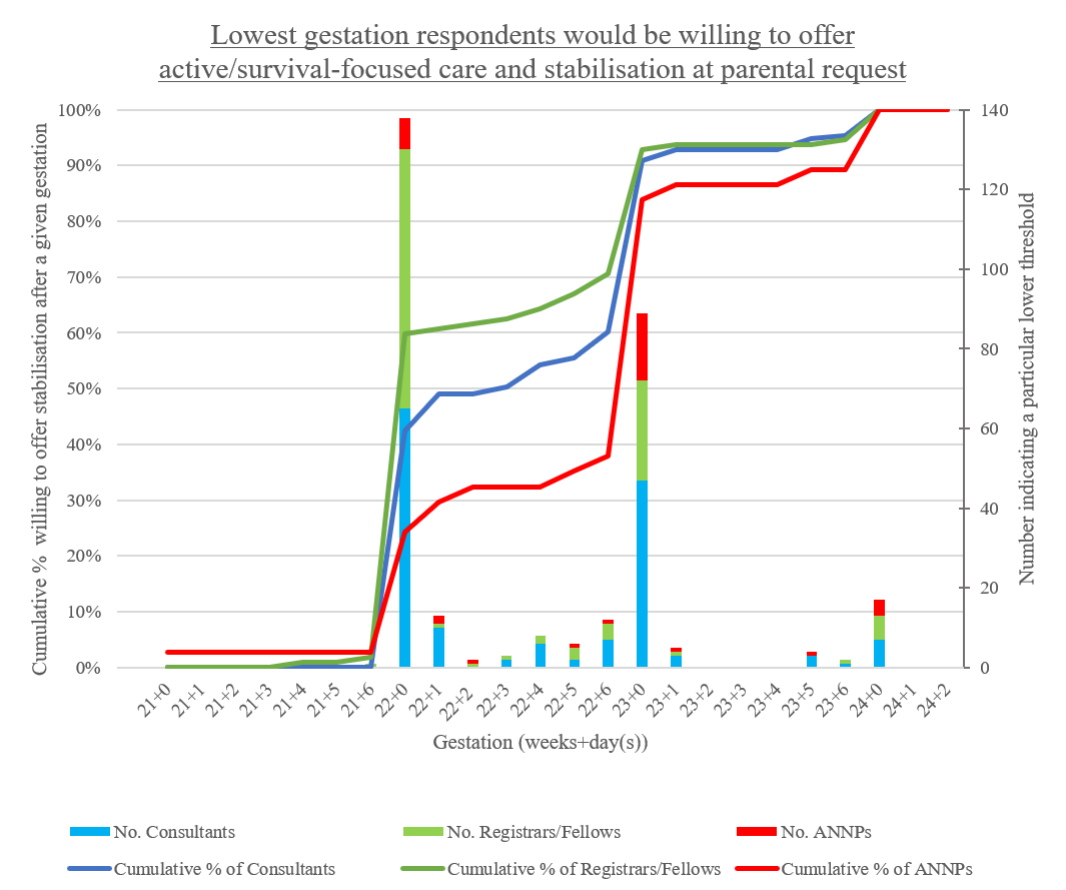
Lowest gestation participants would be willing to offer active/survival-focused care and stabilisation (Active Treatment) at parental request (n=303). One consultant was not willing to select a gestational age with the information provided thus was excluded. One ANNP indicated that they would always offer Active Treatment at parental request. The line graph shows the cumulative percentage, indicating the proportion of respondents prepared to provideActive Treatment at a given gestation if parents request it. The stacked bar graph indicates the number of respondents who selected a particular lower threshold. For example, 47 consultants selected a lower threshold of 23^+0^ weeks, and 91% of consultants were willing to provide Active Treatment for an infant born at 23^+0^ weeks. ANNP, advanced neonatal nurse practitioner.

When asked about the lowest gestation they would offer Active Treatment at parental request, responses ranged from 21^+4^ to 24^+0^ weeks (median lower threshold 22^+1^ weeks) (figure 1), while one participant indicated always resuscitating a liveborn infant if requested. Sixty per cent of respondents stated a lower limit between 22^+0^ and 22^+6^ weeks (n=182), with 33% indicating a gestation between 23^+0^ and 23^+6^ weeks (n=100). Eighty-one per cent of chosen gestational ages corresponded with the beginning of a gestational week (ie, 22^+0^/23^+0^/24^+0^).

Registrars/fellows were most likely to stabilise/resuscitate between 22^+0^ and 22^+6^ weeks (figure 1) with 58% (n=65) selecting a gestation of 22^+0^, compared with 42% of consultants (n=65, Relative Risk (RR)=1.37, 95% CI 1.07 to 1.74, p=0.01) and 22% of ANNPs (n=8, RR=2.68, 95% CI 1.42 to 5.06, p<0.01).

Respondents working in NICU were almost twice as likely to select a gestation of 22^+0^ weeks, compared with those working in other centres (58% vs 31%, RR=1.86, 95% CI 1.18 to 2.94, p=0.01) (online supplemental figure 2). When analysing NICU-workers alone (n=158), registrars/fellows were 1.45 times as likely to choose gestations of 22^+0^ weeks compared with consultants and ANNPs (70% vs 48%, 95% CI 1.11 to 1.89, p=0.01).

#### The upper threshold

The highest gestation at which respondents would offer Palliative Care at parental request was 23^+6^/24^+0^ weeks for 59% of those surveyed (n=172). Twenty-one per cent of respondents would withhold resuscitation up to 24^+6/^25^+0^ weeks (n=62). Four respondents (1 consultant, 2 ANNPs and 1 other) were unwilling to withhold resuscitation at any gestation and 10 had no upper limit. Eighty-seven per cent of chosen gestational ages corresponded with the last day or beginning of a gestational week (eg, 23^+6^/24^+0^).

**Figure 2:**
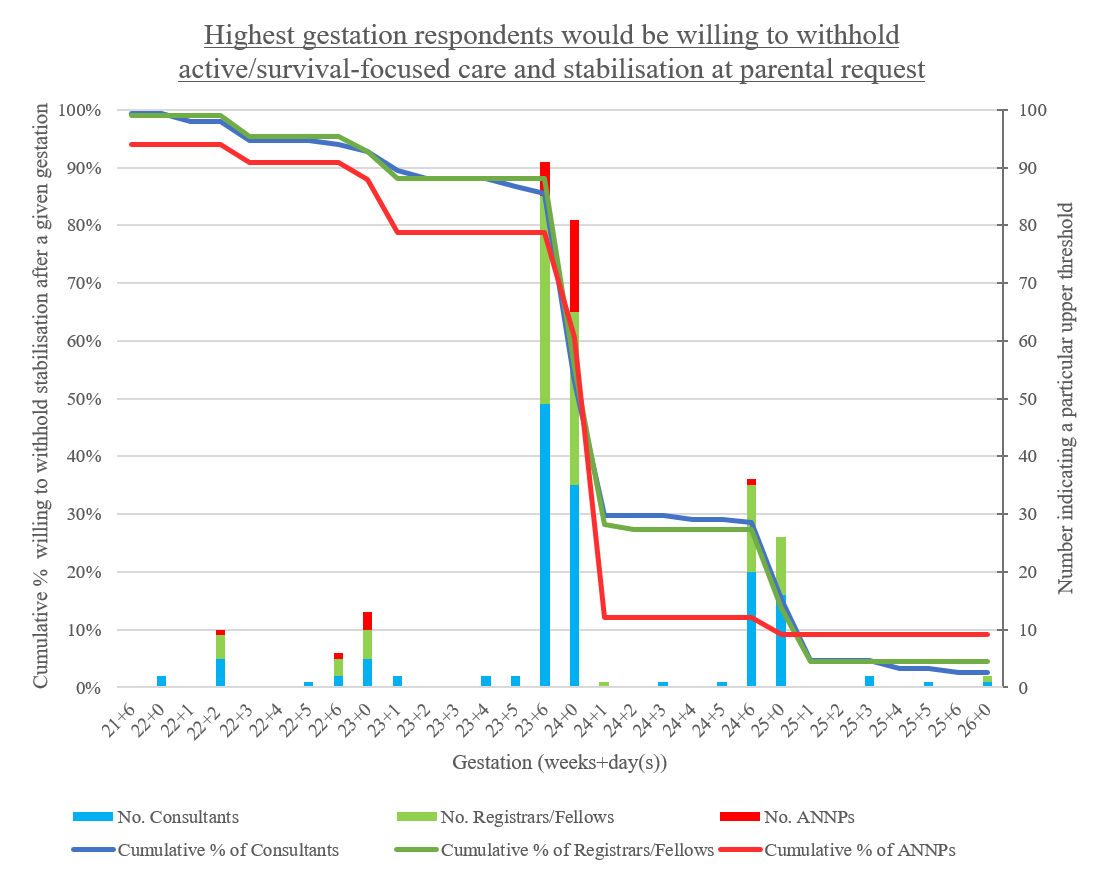
Highest gestation participants would be willing to withhold active/survival-focused care and stabilisation (Active Treatment) at parental request (n=294). Two consultants were not willing to select a gestational age with the information provided thus were excluded. Four respondents indicated never being willing to withhold Active Treatment at parental request, while 10 indicated that they had no upper limit for withholding Active Treatment. The line graph shows the cumulative percentage, indicating the proportion of respondents prepared to withhold Active Treatment at a given gestation if parents refused it. The stacked bar graph indicates the number of respondents who selected a particular upper threshold. For example, 35 consultants selected an upper threshold of 24^+0^ weeks, and 53% of consultants were willing to withhold Active Treatment for an infant born at 24^+0^ weeks.ANNP, advanced neonatal nurse practitioner.

There was a similar pattern of upper limits regardless of professional group or type of unit (figure 2 and online supplemental figure 3).

Across the two questions, two participants indicated feeling unable to respond without additional information including *‘*antenatal setting, antenatal steroids (and fetal) sex*’*.

### Viability

Almost two-thirds of respondents understood the concept of ‘fetal viability’ to reflect possibility of survival after birth at a particular gestation (63%); others indicated that that the majority (25%) or vast majority of infants must survive (7%) (table 2). One respondent wrote that they never use the term ‘viable’ in their practice.

**Table 2:** Respondents’ selections of conceptual elements of a definition of ‘viability’

Concept	Whether or not a fetus or newborn is considered viable at a particular gestation depends on…	Respondents,* n (%)
The proportion of infants who survive	It is possible for infants to survive if born at this gestation	129 (63.2)
	The majority (>50%) of infants born at this gestation will survive	52 (25.5)
	The vast majority (>80%) of infants born at this gestation will survive	15 (7.4)
	Other	8 (3.9)
Survival with or without disability	Without disability	8 (3.9)
	Without severe disability	70 (34.3)
	With or without disability	122 (59.8)
	Other	4 (2.0)
Survival with or without medical intervention	Without medical intervention	4 (2.0)
	With medical interventions that are currently accessible to the infant and the treating team	120 (58.8)
	With medical interventions that could keep the fetus alive, even if they are not accessible to the infant	75 (36.8)
	Other	5 (2.5)

*Total number of respondents who answered each question=204.

Sixty per cent of participants indicated that likelihood of disability was irrelevant to viability, while some suggested viability should reflect survival without severe disability (34%) or without any disability (4%). One participant wrote that the defining factor was ‘Whether it will yield this infant and their family an acceptable quality of life’.

Almost two-thirds of respondents (59%) thought viability reflected survival with medical interventions available to the treating team, while 37% indicated that interventions need not be currently accessible. Comments included that the interventions may not be currently accessible, but should be potentially accessible (eg, within that country) (n=2), viability reflected survival without medical intervention (n=4) and viability was associated with presence of a heart rate (presumably prior to medical intervention) (n=2).

**Figure 3:**
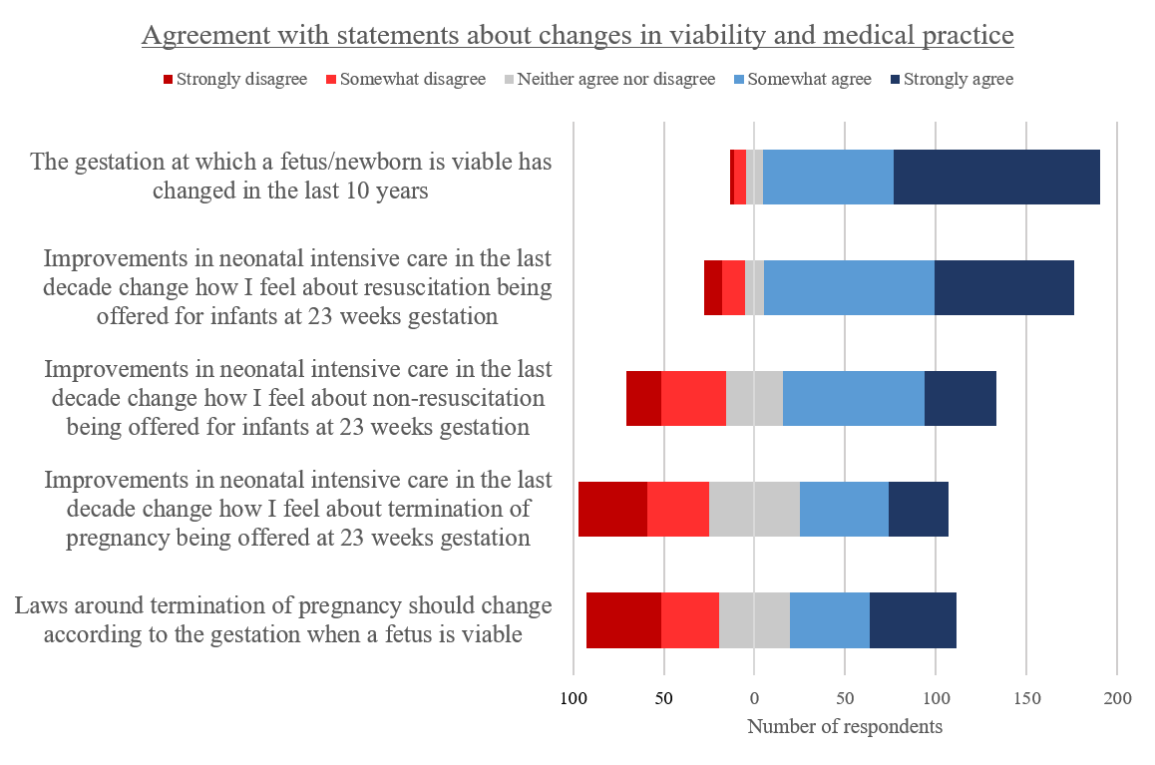
Level of agreement with statements regarding how viability and medical practices change over time (n=204).

The vast majority of respondents (91%) somewhat or strongly agreed that the gestation at which an infant is considered viable had changed in the last decade (figure 3). Most respondents also agreed that improvements in neonatal intensive care over the last decade changed how they felt about resuscitation (Active Treatment) (84%) or non-resuscitation (Palliative Care) (58%) of infants born at 23 weeks of gestation.

Respondents were divided in their views about the impact of advances in neonatal treatment in the past decade on abortion. Forty per cent indicated such advances changed how they felt about abortion at 23 weeks, while 35% indicated unchanged views. Forty-five per cent agreed that abortion laws should change according to the gestation when a fetus is viable, while 36% disagreed, and 19% neither agreed nor disagreed.

Views on viability were similar between professional groups (online supplemental figure 4).

## Discussion

This survey assessed views of UK neonatal consultants, registrars/fellows and ANNPs on decision-making around the care of EPIs. We found that in 2020, UK neonatal staff involved in decisions around Active Treatment reported a median grey zone for Active Treatment between 22^+1^ and 24^+0^ weeks. Registrars/fellows, and those working in NICUs, were more likely to select a lower threshold gestation of 22^+0^ compared with other groups.

### The grey zone

The median lower threshold of 22^+1^ weeks for consideration of Active Treatment suggests a shift in views and practice: in 2008 and 2016 only a minority of UK neonatologists would resuscitate prior to 23^+0^ weeks.^14 15 20^

Survey results suggest that the UK lower threshold is now earlier than some comparable countries. A 2019 review including 15 studies found the majority of physicians would not resuscitate at 22 weeks (85%–100%) and 23 weeks (57%–82%).^21^ However, a lower threshold of 22 weeks has been reported from centres in countries such as Sweden, USA and Japan.^15 22–24^

Changing attitudes of UK clinicians is likely to have been influenced by the publication of the 2019 revised BAPM framework, which advises that Active Treatment may be considered from 22^+0^ weeks following risk assessment and parental discussions. However, revisions to the framework were partly motivated by emerging international evidence regarding improved survival rates and long-term outcomes for the most immature EPIs^17^ and data indicating that practice was already shifting. According to figures from the maternal, newborn and infant clinical outcome review programme (MBRRACE-UK),^25^ in 2016 23% of babies born alive before 23^+0^ weeks’ gestation in the UK were receiving Active Treatment.

In our survey, registrars/fellows indicated earlier gestational thresholds for Active Treatment than their colleagues. This may be partly explained by clinical practice location: NICU-based respondents were almost twice as likely to select a lower threshold of 22^+0^ weeks and registrars/fellows were largely based in NICUs. This finding is consistent with the 2019 BAPM framework, which encourages consideration of delivery location in risk assessment and decision-making, in the knowledge that EPIs have improved outcomes if delivered in a tertiary obstetric centre with a colocated NICU.^17^

Another explanation may relate to consultants’ and ANNPs’ additional professional experience. This could facilitate comfort-focused care provision, given it may require more demanding decision-making and communication. Alternatively, exposure to adverse outcomes, or familiarity with prior guidance, might make experienced clinicians more reluctant to change practice.

### The upper threshold

Almost two-thirds of respondents selected 23^+6^/24^+0^ weeks as their upper threshold. This is unchanged from UK doctors in 2016.^15^ It is similar, but slightly lower than studies from other high-income countries where participants identified the upper threshold as a point between 24^+0^ and 25^+6^ weeks.^18 26–28^ The lack of change in the upper threshold implies that the grey zone in the UK has widened rather than simply shifted downwards in gestation. This could reflect the greater emphasis in the revised BAPM framework on factors other than just gestational age.

### Understanding of viability

Survey participants understood viability similarly to neonatologists and obstetricians in a recent survey from Victoria, Australia^18^ with respect to the possibility of survival at a given gestation, regardless of disability, with the use of medical interventions accessible to the treating team. A minority of clinicians in both surveys indicated that viability depended on survival without disability.

Most respondents agreed that the gestation at which a fetus is considered viable has changed in the last decade, and that neonatal care advances have changed how they feel about Active Treatment for EPIs. There was ambivalence regarding whether these advances impacted on how participants felt about Palliative Care at the same gestation. These views are consistent with the change and lack of change, respectively, in lower and upper thresholds described above.

Two questions related to abortion. In our survey, there was no consensus among respondents; almost equal proportions agreed and disagreed that improvements in neonatal care influenced their views on abortion, or that law should change. This contrasts somewhat with results from the aforementioned Australian survey, in which obstetricians and neonatologists were more likely to disagree with both statements.^18^

### Limitations

While the survey was modest in sample size, in 2015 there were 466 NHS specialists in neonatal medicine and 189 general paediatricians with a special interest in neonatology in the UK^29^; the survey therefore may represent one-quarter of consultants working in NHS neonatal care (n=167). Low response rates to online surveys likely reflect high workload and survey fatigue.^30^ It is possible that, in addition, the timing of the survey coinciding with the COVID-19 pandemic may have contributed.

While almost 80% of respondents worked in NICUs and only 30% of neonatal units in the UK were designated NICUs in 2015,^29^ the majority of EPIs are cared for in such units.

Obstetricians, senior midwives and parents all play critical roles in decision-making around the borderline of viability; it would be useful to include their views in further research.

Surveys may not accurately reflect clinical practice. Upper and lower thresholds were determined via a hypothetical case of an infant in a fair condition, while actual clinical decisions would incorporate a broader range of prognostic factors specific to the particular pregnancy and setting, and would be made in multidisciplinary teams, considering parental views.^17 31^

## Conclusions

This paper sought to explore the views of UK senior neonatal clinicians on questions relating to the provision of Active Treatment in the grey zone of fetal viability. We have found a shift in views regarding the lower threshold for Active Treatment from 23^+0^ to 22^+1^ weeks’ gestation, but no change to the upper threshold. There were some differences in the attitudes to decisions between clinicians of different grades, and those working in NICUs compared with other types of neonatal units.

Although this paper has focused on the role of gestational age in decision-making, current UK guidelines recommend an individualised approach. Further research is needed to help understand how UK perinatal clinicians incorporate other risk factors and parental views into decisions about treatment of EPIs.

## Data Availability

Data are available in a public, open access repository. The data will be made available via the Open Science Framework after publication.
