# Supplementary material for: Viability and thresholds for treatment of extremely preterm infants: survey of UK neonatal professionals
Source: Arch Dis Child Fetal Neonatal Ed. 2021 Apr 29;106(6):596–602. doi: 10.1136/archdischild-2020-321273 (PMC8543207; doi:10.1136/archdischild-2020-321273)
Supplement: Supplementary data [file archdischild-2020-321273supp001.pdf]

Supplement 1 – Online Survey

Viable Views: Thresholds for Resuscitation of Extremely Preterm Infants in the UK 2020

Start of Block: Explanatory Statement and Consent Form

Explanatory Statement and Consent Form

Oxford Uehiro Centre for Practical Ethics, Littlegate House, St Ebbes St, Oxford OX1 1PT, Tel 01865 286 888

Study title: Viable Views: Thresholds for Resuscitation of Extremely Preterm Infants in the UK 2020

General Information

The aim of this study is to determine the views of UK doctors (consultants and registrars) and nurse practitioners (ANNPs) who are involved in the decision-making around the care of extremely preterm infants on ethical questions relating to thresholds for resuscitation/stabilisation of extremely premature infants.

We appreciate your interest in participating in this online survey. You have been invited to participate as your professional practice involves decision-making around the care of extremely preterm infants. Please read through this information carefully before agreeing to participate by ticking the ‘yes’ box below.

You may ask any questions before deciding to take part by contacting the researcher (details below).

The survey asks you to consider scenarios relating to the management of extremely premature infants born in various conditions. It should take about 10 minutes.

Do I have to take part? Please note that your participation is voluntary. If you do decide to take part, you may withdraw at any point during the questionnaire for any reason before submitting your answers by pressing the ‘Exit’ button/closing the browser.

How will my data be used? Your answers will be completely anonymous, and we will take all reasonable measures to ensure that they remain confidential. (Contact details for the prize

draw will be stored separately to survey results to preserve anonymity. It will be stored on secure Qualtrics servers and deleted after the draw has been completed.)

Your anonymous data will be stored in a password-protected file and may be used in academic publications. Your IP address will not be stored. Research data will be stored for a minimum of three years after publication or public release. The anonymous data that we collect from you may be transferred to, stored and/or processed at a destination outside the UK and the European Economic Area ("EEA").

Who will have access to my data? Qualtrics is the data controller with respect to your personal data and, as such, will determine how your personal data is used. Please see their privacy statement here. Qualtrics will share only fully anonymised data with the University of Oxford, for the purposes of research.

Summaries of (anonymous) research results may be shared with the British Association of Perinatal Medicine.

Responsible members of the University of Oxford may be given access to data for monitoring and/or audit of the study to ensure we are complying with guidelines, or as otherwise required by law.

The Principal Researcher is Professor Dominic Wilkinson, who is attached to the Oxford Uehiro Centre for Practical Ethics at the University of Oxford.

Other researchers include Dr Katherine Wood (Oxford University Hospitals NHS Foundation Trust), and Lydia Di Stefano (Monash University, Australia)

This project has been reviewed by, and received ethics clearance through, the University of Oxford Central University Research Ethics Committee [R69766/RE001].

Who do I contact if I have a concern about the study or I wish to complain? If you have a concern about any aspect of this study, please speak to Professor Dominic Wilkinson (Dominic.wilkinson@philosophy.ox.ac.uk), and we will do our best to answer your query. I will acknowledge your concern within 10 working days and give you an indication of how it will be dealt with.

If you remain unhappy or wish to make a formal complaint, please contact the Chair of the Research Ethics Committee at the University of Oxford who will seek to resolve the matter as soon as possible:
Chair, Medical Sciences Interdivisional Research Ethics Committee;
Email: ethics@medsci.ox.ac.uk
Address: Research Services, University of Oxford, Wellington Square, Oxford OX1 2JD

To enter the draw for free entry to the BAPM annual conference 2021, please follow the link to enter your email address at end of the survey.

Page Break

X→

If you have read the information above and agree to participate with the understanding that the data (including any personal data) you submit will be processed accordingly, please check the relevant box below to get started.

- ◯ Yes (1)
- ◯ No (2)

Skip To: End of Survey If If you have read the information above and agree to participate with the understanding that the d... = No

Page Break

X→

Does your professional practice involve decision-making around the care of extremely preterm infants (eg consultant or registrar in neonatology or an advanced neonatal nurse practitioner who attends preterm births independently)?

- ◯ Yes (1)
- ◯ No (2)

Display This Question:

If Does your professional practice involve decision-making around the care of extremely preterm infa... = No

X→

You have indicated that your professional practice does not involve decision-making around the care of extremely preterm infants. Was this an error?

By selecting 'No', you will be taken to the end of the survey.

- ◯ Yes (1)
- ◯ No (2)

Skip To: End of Survey If You have indicated that your professional practice does not involve decision-making around the ca... = No

End of Block: Explanatory Statement and Consent Form

Start of Block: Professional Level

What is your professional level?

- ○ Registrar (1)
- ○ Consultant (2)
- ○ ANNP (3)
- ○ Other (4)

End of Block: Professional Level

Start of Block: Section 1

SECTION 1

For **all** the following questions, please assume that an early dating scan has previously been performed to estimate gestational age, and that there are no other known medical conditions affecting the fetus or mother.
"Active Resuscitation" in these scenarios refers to resuscitation/stabilisation of the extremely preterm infant - eg provision of respiratory support including intubation and other measures as appropriate.

Page Break

A mother has gone into extremely premature labour.

She has been counselled and received accurate information about the chance of survival if the infant is born in the next 24 hours, about potential complications of neonatal intensive care, and about the risk of long-term neurodevelopmental problems if the infant survives.

Imagine that the infant is **born in fair condition**. Assume that any other **risk factors are average** or as expected for this gestation.

X→

1) If the parents have requested active resuscitation, what is the lowest gestational age at which you would be prepared to attempt resuscitation (including intermittent positive pressure ventilation and intubation)? (i.e. if the infant is more premature than this you would not resuscitate).

Please select from the following dropdown list:
- Select 'Other' if you would like to specify a gestation <21 or >/=26 weeks.
- Select 'NA' if you do not have a set lower-limit (i.e. you would always attempt resuscitation if requested and the infant is born alive).

○ NA - I would always attempt resuscitation if requested & the infant is born alive (1)

○ Other i.e. < 21 weeks or >/= 26 weeks (2)

○ 21+0 (3)

○ …

○ 25+6 (14)

Display This Question:

If 1) If the parents have requested active resuscitation, what is the lowest gestational age at whic... = Other i.e. < 21 weeks or >/= 26 weeks

If other gestation, please specify, e.g. X weeks and Y days.

____

X→

2) If the parents have requested non-resuscitation, what is the highest gestational age at which you would be prepared to NOT attempt resuscitation (including intermittent positive pressure ventilation and intubation)? (i.e. if the infant is more mature than this you would resuscitate against parental wishes).

Please select from the following dropdown list:
- Select 'Other' if you would like to specify a gestation <21 or >/=26 weeks.
- Select 'No upper limit' if you do not have a set upper-limit (i.e. you would always be willing

to NOT attempt resuscitation if requested).
- Select 'Always attempt resus' if you would always attempt resuscitation, even if against parental wishes, if the infant was born alive.

◯ No upper limit (0)

◯ Always attempt resus (1)

◯ Other i.e. < 21 weeks or >/= 26 weeks (2)

◯ 21+0 (3)

◯ …

◯ 25+6 (14)

Display This Question:

If 2) If the parents have requested non-resuscitation, what is the highest gestational age at which... = Other i.e. < 21 weeks or >/= 26 weeks

If other gestation, please specify, e.g. X weeks and Y days.

____

End of Block: Section 1

SECTION 2

This section contained questions regarding the British Association of Perinatal Medicine's publication *Perinatal management of extreme preterm birth before 27 weeks of gestation: a framework for practice*. Respondents were asked to consider scenarios with extremely premature infants where risk factors (e.g. antenatal steroids, fetal sex, fetal growth, neonatal unit type) as well as their gestational ages varied. The results of these questions will be presented in a subsequent paper.

Start of Block: Section 3

SECTION 3

Medical and professional guidelines relating obstetric and neonatal management often refer to fetuses or infants as "viable" or "non-viable".

In trying to understand whether a fetus or newborn infant is viable, there are multiple aspects that need to be considered.

We are interested in what you understand by this term.

X→

1) **Medical intervention:** Thinking about whether or not a fetus or newborn is viable depends on whether the fetus or newborn can survive...

- ○ Without medical intervention (1)
- ○ With medical interventions that are currently accessible to the infant and the treating team (2)
- ○ With medical interventions that could keep the fetus alive, even if they are not accessible to the infant and the treating team (e.g. treatment that is available in a more specialised centre, or in another country) (3)
- ○ Other (4)

Display This Question:

If 1) Medical intervention: Thinking about whether or not a fetus or newborn is viable depends on wh... = Other

If other, please provide more detail.

____

X→

Di Stefano LM, *et al. Arch Dis Child Fetal Neonatal Ed* 2021; 106:596–602. doi: 10.1136/archdischild-2020-321273

2) **The proportion of infants who survive:** Thinking about whether or not a fetus or newborn is considered viable at a particular gestation depends on whether...

- It is possible for infants to survive if born at this gestation (1)
- The majority (>50%) of infants born at this gestation will survive (2)
- The vast majority (>80%) of infants born at this gestation will survive (3)
- Other (4)

Display This Question:

If 2) The proportion of infants who survive: Thinking about whether or not a fetus or newborn is con... = Other

If other, please provide more detail.

____

X→

3) **Survival with or without disability:** Thinking about whether or not a fetus or newborn is considered viable at a particular gestation depends on the proportion of infants that survive...

- Without disability (1)
- Without severe disability (2)
- With or without disability (3)
- Other (4)

Display This Question:

If 3) Survival with or without disability: Thinking about whether or not a fetus or newborn is consi... = Other

If other, please provide more detail.

____

Page Break

4) Please indicate how much you agree or disagree with the following statements.

	Strongly agree (1)	Somewhat agree (2)	Neither agree nor disagree (3)	Somewhat disagree (4)	Strongly disagree (5)
The gestation at which a fetus/newborn is viable has changed in the last 10 years (1)	○	○	○	○	○
Improvements in neonatal intensive care in the last decade change how I feel about resuscitation being offered for infants at 23 weeks gestation (2)	○	○	○	○	○
Improvements in neonatal intensive care in the last decade change how I feel about non-resuscitation being offered for infants at 23 weeks gestation (3)	○	○	○	○	○
Improvements in neonatal intensive care in the last decade change how I feel about termination of pregnancy being offered at 23 weeks gestation (4)	○	○	○	○	○
Laws around termination of pregnancy should change according to the gestation when a fetus is viable (5)	○	○	○	○	○

End of Block: Section 3 Understanding Viability

Start of Block: Section 4 Personal Data

SECTION 4

The following questions are to help in our interpretation of survey results, but are optional.
Gender

- Male (1)
- Female (2)
- Other (3)

Age (years)

- 18-30 (1)
- 31-40 (2)
- 41-50 (3)
- 51-60 (4)
- 61-70 (5)
- 71+ (6)

For how many years have you worked with extremely premature infants?

- 0-3 (1)
- 4-7 (2)
- 8-15 (3)
- 16+ (4)

Di Stefano LM, *et al. Arch Dis Child Fetal Neonatal Ed* 2021; 106:596–602. doi: 10.1136/archdischild-2020-321273

X→

What type of neonatal centre do you usually work in?

- NICU (level 3/tertiary centre) (1)
- LNU Local Neonatal Unit (4)
- SCU Special Care Unit (5)
- Other (6)

End of Block: Section 4 Personal Data

Start of Block: Thank you

Thank you for completing this survey. All information that you have provided will be kept anonymous. If you would like to be entered in the draw to win FREE REGISTRATION to the BAPM Annual Conference in 2021, please click on the link here. This will take you to a separate page where you can enter your email address.

End of Block: Thank you

Supplement 2– Supplemental Figures and Tables

Supplemental Figures

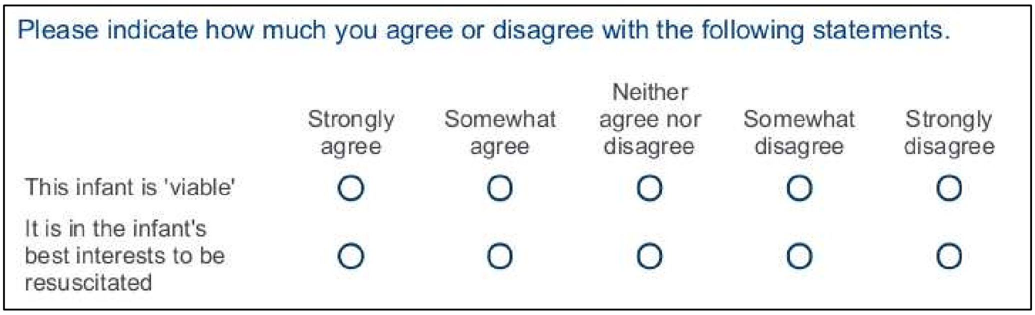

Please indicate how much you agree or disagree with the following statements.

	Strongly agree	Somewhat agree	Neither agree nor disagree	Somewhat disagree	Strongly disagree
This infant is 'viable'	O	O	O	O	O
It is in the infant's best interests to be resuscitated	O	O	O	O	O

Supplemental Figure 1. Example of Likert scale question.

Di Stefano LM, *et al. Arch Dis Child Fetal Neonatal Ed* 2021; 106:596–602. doi: 10.1136/archdischild-2020-321273

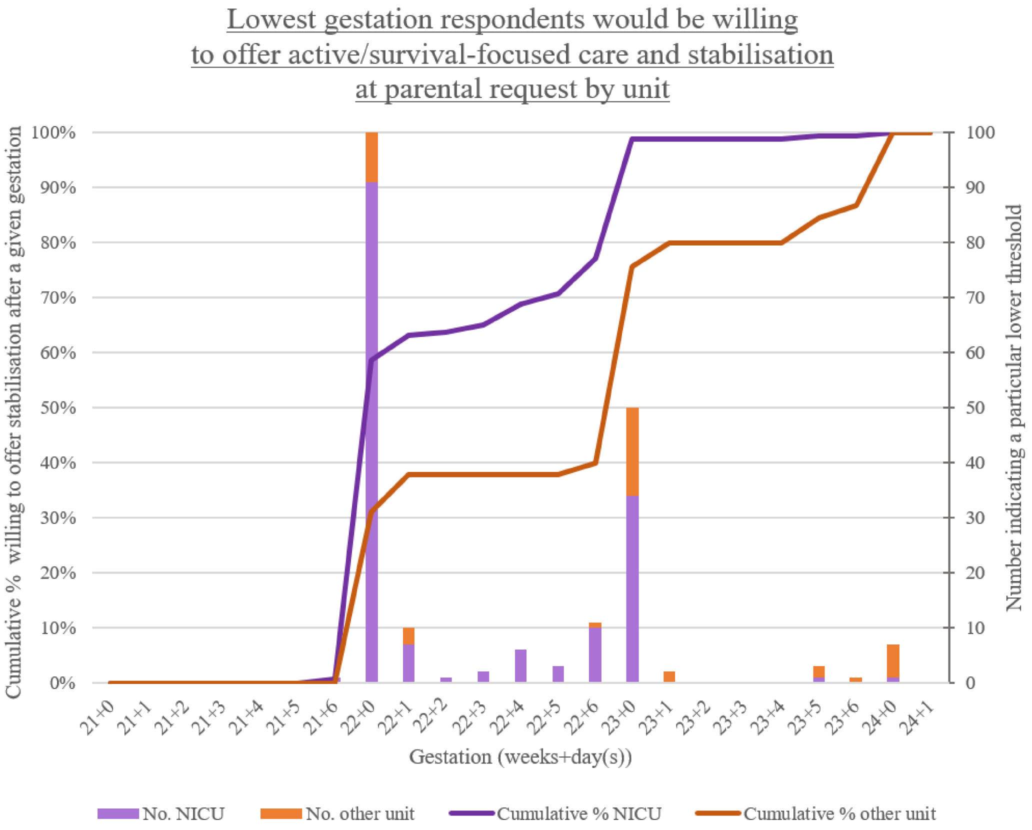

Supplemental Figure 2. Lowest gestation participants would be willing to offer active/survival-focused care and stabilisation (Active Treatment) at parental request by unit (n=202).

The line graph shows the cumulative percentage, indicating the proportion of respondents prepared to provide Active Treatment at a given gestation if parents request it. The stacked bar graph indicates the number of respondents who selected a particular lower threshold. For example, 34 NICU clinicians selected a lower threshold of 23^{+0} weeks, and 99% were willing to provide Active Treatment for an infant born at 23^{+0} weeks**.**

Di Stefano LM, *et al. Arch Dis Child Fetal Neonatal Ed* 2021; 106:596–602. doi: 10.1136/archdischild-2020-321273

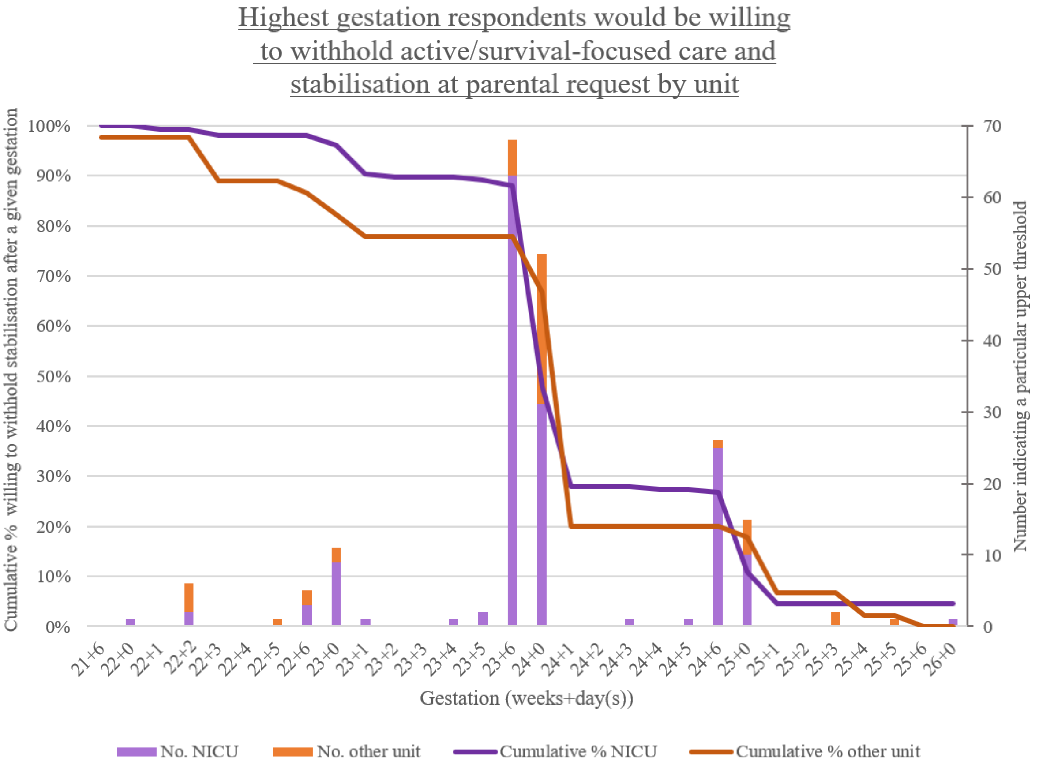

Supplemental Figure 3. Highest gestation participants would be willing to withhold active/survival-focused care and stabilisation (Active Treatment) at parental request by unit (n=202).

The line graph shows the cumulative percentage, indicating the proportion of respondents prepared to withhold Active Treatment at a given gestation if parents refused it. The stacked bar graph indicates the number of respondents who selected a particular upper threshold. For example, 31 NICU staff selected an upper threshold of 24^{+0} weeks, and 48% were willing to withhold Active Treatment for an infant born at 24^{+0} weeks.

Di Stefano LM, *et al. Arch Dis Child Fetal Neonatal Ed* 2021; 106:596–602. doi: 10.1136/archdischild-2020-321273

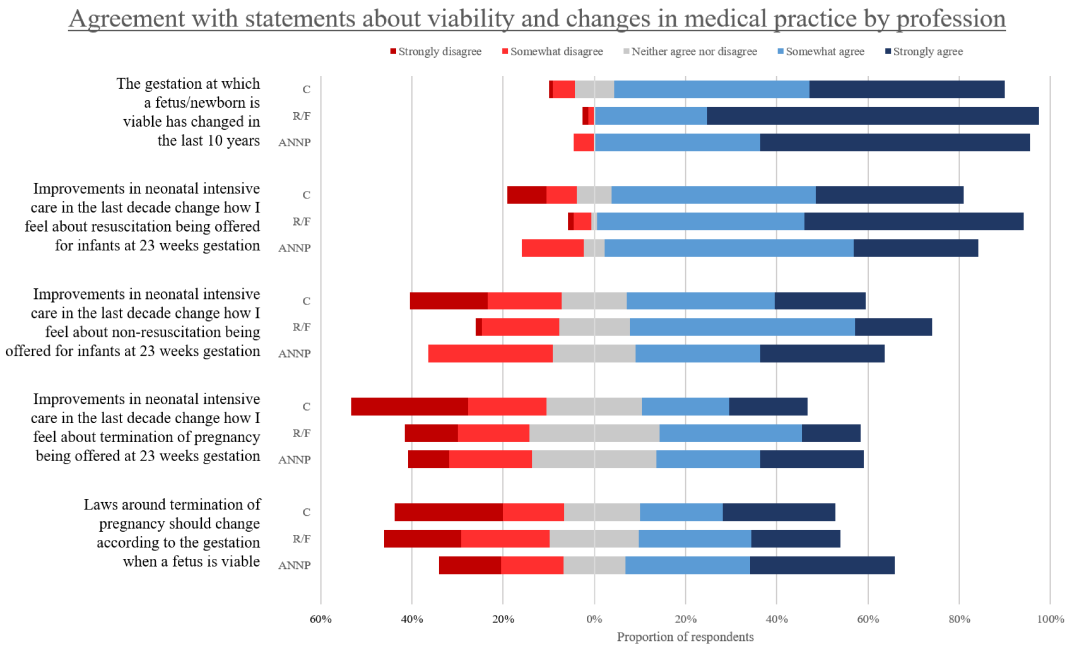

Supplemental Figure 4. Level of agreement with statements regarding how viability and medical practices change over time by professional level n=204.

C= consultants, R/F= registrars/fellows, ANNP= advanced neonatal nurse practitioners

Di Stefano LM, *et al. Arch Dis Child Fetal Neonatal Ed* 2021; 106:596–602. doi: 10.1136/archdischild-2020-321273

Supplemental Tables - Data From Which Figures Are Based

Supplemental Table 1. Figure 1 Numerical Data

	**Consultants**		**Registrars/Fellows**		**ANNPs**	
**Gestation**	**No.**	**Cumulative %**	**No.**	**Cumulative %**	**No.**	**Cumulative %**
Always offer Active Treatment i.e. 0+0*	0	0.00%	1	0.00%	1	2.70%
21+0	0	0.00%	0	0.00%	0	2.70%
21+1	0	0.00%	0	0.00%	0	2.70%
21+2	0	0.00%	0	0.00%	0	2.70%
21+3	0	0.00%	0	0.00%	0	2.70%
21+4	0	0.00%	1	0.89%	0	2.70%
21+5	0	0.00%	0	0.89%	0	2.70%
21+6	0	0.00%	1	1.79%	0	2.70%
22+0	65	42.48%	65	59.82%	8	24.32%
22+1	10	49.02%	1	60.71%	2	29.73%
22+2	0	49.02%	1	61.61%	1	32.43%
22+3	2	50.33%	1	62.50%	0	32.43%
22+4	6	54.25%	2	64.29%	0	32.43%
22+5	2	55.56%	3	66.96%	1	35.14%
22+6	7	60.13%	4	70.54%	1	37.84%
23+0	47	90.85%	25	92.86%	17	83.78%
23+1	3	92.81%	1	93.75%	1	86.49%
23+2	0	92.81%	0	93.75%	0	86.49%
23+3	0	92.81%	0	93.75%	0	86.49%
23+4	0	92.81%	0	93.75%	0	86.49%
23+5	3	94.77%	0	93.75%	1	89.19%
23+6	1	95.42%	1	94.64%	0	89.19%
24+0	7	100.00%	6	100.00%	4	100.00%
24+1	0	100.00%	0	100.00%	0	100.00%
24+2	0	100.00%	0	100.00%	0	100.00%
24+3	0	100.00%	0	100.00%	0	100.00%
24+4	0	100.00%	0	100.00%	0	100.00%
24+5	0	100.00%	0	100.00%	0	100.00%
24+6	0	100.00%	0	100.00%	0	100.00%
**Total**	153		113		37	

* Not within graph axes

Di Stefano LM, *et al. Arch Dis Child Fetal Neonatal Ed* 2021; 106:596–602. doi: 10.1136/archdischild-2020-321273

Supplemental Table 2. Figure 2 Numerical Data

	**Consultants**		**Registrars/Fellows**		**ANNPs**	
**Gestation**	**No.**	**Cumulative %**	**No.**	**Cumulative %**	**No.**	**Cumulative %**
Never willing to withhold Active Treatment i.e. 0+0*	1	100.00%	1	100.00%	2	100.00%
21+0	0	99.34%	0	99.09%	0	93.94%
21+1	0	99.34%	0	99.09%	0	93.94%
21+2	0	99.34%	0	99.09%	0	93.94%
21+3	0	99.34%	0	99.09%	0	93.94%
21+4	0	99.34%	0	99.09%	0	93.94%
21+5	0	99.34%	0	99.09%	0	93.94%
21+6	0	99.34%	0	99.09%	0	93.94%
22+0	2	99.34%	0	99.09%	0	93.94%
22+1	0	98.01%	0	99.09%	0	93.94%
22+2	5	98.01%	4	99.09%	1	93.94%
22+3	0	94.70%	0	95.45%	0	90.91%
22+4	0	94.70%	0	95.45%	0	90.91%
22+5	1	94.70%	0	95.45%	0	90.91%
22+6	2	94.04%	3	95.45%	1	90.91%
23+0	5	92.72%	5	92.73%	3	87.88%
23+1	2	89.40%	0	88.18%	0	78.79%
23+2	0	88.08%	0	88.18%	0	78.79%
23+3	0	88.08%	0	88.18%	0	78.79%
23+4	2	88.08%	0	88.18%	0	78.79%
23+5	2	86.75%	0	88.18%	0	78.79%
23+6	49	85.43%	36	88.18%	6	78.79%
24+0	35	52.98%	30	55.45%	16	60.61%
24+1	0	29.80%	1	28.18%	0	12.12%
24+2	0	29.80%	0	27.27%	0	12.12%
24+3	1	29.80%	0	27.27%	0	12.12%
24+4	0	29.14%	0	27.27%	0	12.12%
24+5	1	29.14%	0	27.27%	0	12.12%
24+6	20	28.48%	15	27.27%	1	12.12%
25+0	16	15.23%	10	13.64%	0	9.09%
25+1	0	4.64%	0	4.55%	0	9.09%
25+2	0	4.64%	0	4.55%	0	9.09%
25+3	2	4.64%	0	4.55%	0	9.09%
25+4	0	3.31%	0	4.55%	0	9.09%
25+5	1	3.31%	0	4.55%	0	9.09%
25+6	0	2.65%	0	4.55%	0	9.09%
26+0	1	2.65%	1	4.55%	0	9.09%
No upper limit*	3	1.99%	4	3.64%	3	9.09%
**Total**	151		110		33	

* Not within graph axes

Di Stefano LM, *et al. Arch Dis Child Fetal Neonatal Ed* 2021; 106:596–602. doi: 10.1136/archdischild-2020-321273

Supplemental Table 3. Figure 3 Numerical Data

Question	Strongly agree		Somewhat agree		Neither agree nor disagree		Somewhat disagree		Strongly disagree	
The gestation at which a fetus/newborn is viable has changed in the last 10 years	55.88%	114	35.29%	72	4.41%	9	3.43%	7	0.98%	2
Improvements in neonatal intensive care in the last decade change how I feel about resuscitation being offered for infants at 23 weeks gestation	37.75%	77	46.08%	94	4.90%	10	6.37%	13	4.90%	10
Improvements in neonatal intensive care in the last decade change how I feel about non-resuscitation being offered for infants at 23 weeks gestation	19.61%	40	38.24%	78	15.20%	31	17.65%	36	9.31%	19
Improvements in neonatal intensive care in the last decade change how I feel about termination of pregnancy being offered at 23 weeks gestation	16.18%	33	24.02%	49	24.51%	50	16.67%	34	18.63%	38
Laws around termination of pregnancy should change according to the gestation when a fetus is viable	23.53%	48	21.57%	44	19.12%	39	15.69%	32	20.10%	41

Supplemental Table 4. Supplemental Figure 2 Numerical Data

**Question**	**No. NICU**	**Cumulative % NICU**	**No. other unit**	**Cumulative % other unit**
21+0	0	0.00%	0	0.00%
21+1	0	0.00%	0	0.00%
21+2	0	0.00%	0	0.00%
21+3	0	0.00%	0	0.00%
21+4	0	0.00%	0	0.00%
21+5	0	0.00%	0	0.00%
21+6	1	0.64%	0	0.00%
22+0	91	58.60%	14	31.11%
22+1	7	63.06%	3	37.78%
22+2	1	63.69%	0	37.78%
22+3	2	64.97%	0	37.78%
22+4	6	68.79%	0	37.78%
22+5	3	70.70%	0	37.78%
22+6	10	77.07%	1	40.00%
23+0	34	98.73%	16	75.56%
23+1	0	98.73%	2	80.00%
23+2	0	98.73%	0	80.00%
23+3	0	98.73%	0	80.00%
23+4	0	98.73%	0	80.00%
23+5	1	99.36%	2	84.44%
23+6	0	99.36%	1	86.67%
24+0	1	100.00%	6	100.00%
24+1	0	100.00%	0	100.00%
**Total**	157		45	

Di Stefano LM, *et al. Arch Dis Child Fetal Neonatal Ed* 2021; 106:596–602. doi: 10.1136/archdischild-2020-321273

Supplemental Table 5. Supplemental Figure 3 Numerical Data

**Question**	**No. NICU**	**Cumulative % NICU**	**No. other unit**	**Cumulative % other unit**
Never willing to withhold Active Treatment i.e. 0+0*	0	100.00%	1	100.00%
21+0*	0	100.00%	0	97.78%
21+1*	0	100.00%	0	97.78%
21+2*	0	100.00%	0	97.78%
21+3*	0	100.00%	0	97.78%
21+4*	0	100.00%	0	97.78%
21+5*	0	100.00%	0	97.78%
21+6	0	100.00%	0	97.78%
22+0	1	100.00%	0	97.78%
22+1	0	99.36%	0	97.78%
22+2	2	99.36%	4	97.78%
22+3	0	98.09%	0	88.89%
22+4	0	98.09%	0	88.89%
22+5	0	98.09%	1	88.89%
22+6	3	98.09%	2	86.67%
23+0	9	96.18%	2	82.22%
23+1	1	90.45%	0	77.78%
23+2	0	89.81%	0	77.78%
23+3	0	89.81%	0	77.78%
23+4	1	89.81%	0	77.78%
23+5	2	89.17%	0	77.78%
23+6	63	87.90%	5	77.78%
24+0	31	47.77%	21	66.67%
24+1	0	28.03%	0	20.00%
24+2	0	28.03%	0	20.00%
24+3	1	28.03%	0	20.00%
24+4	0	27.39%	0	20.00%
24+5	1	27.39%	0	20.00%
24+6	25	26.75%	1	20.00%
25+0	10	10.83%	5	17.78%
25+1	0	4.46%	0	6.67%
25+2	0	4.46%	0	6.67%
25+3	0	4.46%	2	6.67%
25+4	0	4.46%	0	2.22%
25+5	0	4.46%	1	2.22%
25+6	0	4.46%	0	0.00%
26+0	1	4.46%	0	0.00%
No upper limit*	6	3.82%	0	0.00%
**Total**	157		45	

* Not within graph axes

Supplemental Table 6. Supplemental Figure 4 Numerical Data

Question	Professional Group	Strongly disagree	Somewhat disagree	Neither agree nor disagree	Somewhat agree	Strongly agree
**Laws around termination of pregnancy should change according to the gestation when a fetus is viable**						
	**ANNP[1]**	14%	14%	14%	27%	32%
	**R/F[2]**	17%	19%	19%	25%	19%
	**C[3]**	24%	13%	20%	18%	25%
**Improvements in neonatal intensive care in the last decade change how I feel about termination of pregnancy being offered at 23 weeks gestation**						
	**ANNP**	9%	18%	27%	23%	23%
	**R/F**	12%	16%	29%	31%	13%
	**C**	26%	17%	21%	19%	17%
**Improvements in neonatal intensive care in the last decade change how I feel about non-resuscitation being offered for infants at 23 weeks gestation**						
	**ANNP**	0%	27%	18%	27%	27%
	**R/F**	1%	17%	16%	49%	17%
	**C**	17%	16%	14%	32%	20%
**Improvements in neonatal intensive care in the last decade change how I feel about resuscitation being offered for infants at 23 weeks gestation**						
	**ANNP**	0%	14%	5%	55%	27%
	**R/F**	1%	4%	1%	45%	48%
	**C**	9%	7%	8%	45%	32%
**The gestation at which a fetus/newborn is viable has changed in the last 10 years**						
	**ANNP**	0%	5%	0%	36%	59%
	**R/F**	1%	1%	0%	25%	73%
	**C**	1%	5%	9%	43%	43%

[1] ANNP = Advanced Neonatal Nurse Practitioners, [2] R/F = Registrars/Fellows, [3] C = Consultants

Di Stefano LM, *et al. Arch Dis Child Fetal Neonatal Ed* 2021; 106:596–602. doi: 10.1136/archdischild-2020-321273
